# Antioxidant Capacities and Polyphenol Contents of Kombucha Beverages Based on Vine Tea and Sweet Tea

**DOI:** 10.3390/antiox11091655

**Published:** 2022-08-25

**Authors:** Adila Saimaiti, Si-Yu Huang, Ruo-Gu Xiong, Si-Xia Wu, Dan-Dan Zhou, Zhi-Jun Yang, Min Luo, Ren-You Gan, Hua-Bin Li

**Affiliations:** 1 Guangdong Provincial Key Laboratory of Food, Nutrition and Health, Department of Nutrition, School of Public Health, Sun Yat-sen University, Guangzhou 510080, China; 2 Research Center for Plants and Human Health, Institute of Urban Agriculture, Chinese Academy of Agricultural Sciences, Chengdu 610213, China

**Keywords:** kombucha, vine tea, sweet tea, tea residues, fermentation, antioxidant activity

## Abstract

Kombucha beverage is commonly prepared by black tea infusion fermentation without tea residues, and possesses various health benefits. In this paper, kombucha beverages of two non-*Camellia sinensis* teas, including vine tea (*Ampelopsis* *grossedentata*) and sweet tea (*Rubus suavissimus*), were studied for the first time. The antioxidant activities and polyphenol contents of kombucha beverages were evaluated by ferric-reducing antioxidant power assay, Trolox equivalent antioxidant capacity assay, and Folin-Ciocalteu method, respectively. In addition, effects of tea residues on antioxidant capacities of kombucha beverages were evaluated. The results showed that kombucha beverages from vine tea and sweet tea possessed strong antioxidant activities (especially vine tea kombucha), and fermentation with tea residues could significantly increase antioxidant capacities (maximum increase of 38%) and total phenolic content (maximum increase of 55%) of two kombucha beverages compared with those without tea residues. Moreover, the sensory evaluations showed that the sensory evaluation scores of kombucha with tea residues could be improved compared with those without tea residues. Furthermore, the concentrations of several bioactive components in the kombucha beverages were detected by high-performance liquid chromatography. These kombucha beverages could be used for prevention of several diseases with related of oxidative stress.

## 1. Introduction

Kombucha is a popular beverage with a slightly acidic and sweet taste, and its consumption has increased in recent years [1]. It contains a variety of health substances, such as probiotics, polyphenols, vitamins, minerals as well as organic acids [2]. Kombucha has many health effects, including antioxidant, anti-inflammatory, antimicrobial, immunomodulatory, and hepatoprotective actions, and so on [3]. Kombucha is conventionally prepared based on black tea with tea fungus (a symbiotic culture of acetic acid bacteria, yeasts and lactic acid bacteria), and many bioactive compounds (such as levan) and bacterial cellulose could be produced, which possessed important potential application in food, drug and biomedical industries [4,5,6,7]. In recent years, in addition to various *Camellia sinensis* teas (such as green tea, white tea, oolong tea and black tea), several studies have used other raw materials as viable substrates to prepare kombucha, including herbs, fruits, food industry by-products, etc [8,9]. These kombucha beverages from non-*Camellia sinensis* teas could have significant biological activities and health benefits because they could contain various bioactive components, such as cinnamaldehyde, eugenol, chlorogenic acid, caffeic acid, 6-gingerol, 6-shogaol, quercetin and vanillic acid [10,11]. Furthermore, a recent study found that the antioxidant capacities and polyphenol contents of kombucha beverages could be significantly increased with tea residue fermentation [12].

Vine tea (*Ampelopsis* *grossedentata*) and sweet tea (*Rubus suavissimus*) are two famous tea-like plants (non-*Camellia sinensis* tea) [13,14]. Vine tea is traditionally used both as medicine and food, and dihydromyricetin (DMY) is the abundant flavonoid and main bioactive component of vine tea [13]. Vine tea has various health effects, including antioxidant, anti-inflammatory, antibacterial, hepatoprotective and anticancer activities, etc [15]. Sweet tea is a non-toxic, highly sweet and low-calorie natural plant, which is commonly used as medicine, food and beverage. Sweet tea has several health benefits, including antioxidant, anti-inflammatory, anti-allergic, anticancer, antihypertensive, and anti-obesity effects, and so on [14,16].

Although vine tea and sweet tea have many health benefits and wide application prospects, kombucha beverages based on them have not been reported in the literature. Therefore, kombucha beverages from vine tea and sweet tea were prepared with or without tea residues in this study, and their antioxidant capacities and polyphenol contents were assessed at different fermentation stages. The antioxidant activities were determined by ferric-reducing antioxidant power (FRAP) assay and Trolox equivalent antioxidant capacity (TEAC) assay, and the total phenolic contents (TPC) was determined by the Folin-Ciocalteu method. In addition, the concentrations of several bioactive components in two kombucha beverages were measured using high-performance liquid chromatography with photodiode array detector (HPLC-PDA) [12,17], including gallic acid, dihydromyricetin, myricetin, gallic acid, chlorogenic acid, catechin, rutin and ellagic acid. These kombucha beverages were studied for the first time, and found to possess strong high antioxidant activities (especially vine tea kombucha), which could be used as functional foods to prevent several diseases with related of oxidative stress.

## 2. Materials and Methods

### 2.1. Plant Sample and Reagents

Vine tea was bought from Enshi, Hubei Province, China, and sweet tea was bought from Jinxiu, Guangxi Province, China.

The 6-hydroxy-2,5,7,8-tetramethylchromane-2-carboxylic acid (Trolox), 2,2′-azinobis (3-ethylbenothiazoline-6-sulfonic acid) diammonium salt (ABTS), 2,4,6-tri (2- pyridyl)-S-triazine (TPTZ), Folin–Ciocalteu’s phenol reagent, and gallic acid were purchased from Sigma-Aldrich (St. Louis, MO, USA). Acetic acid, hydrochloric acid, potassium peroxydisulfate, iron(II) sulfate heptahydrate, iron(III) chloride hexahydrate and sodium acetate were bought from Tianjin Chemical Factory (Tianjin, China). Sodium carbonate was bought from Shanghai Yuanye Biological Technology Co., LTD. (Shanghai, China). Methanol, sucrose and formic acid were bought from Macklin Chemical Factory (Shanghai, China). The standard compounds gallic acid, chlorogenic acid, catechin, epicatechin, ellagic acid, rutin, myricetin, quercitrin, quercetin, astragalin, and kaempferol for HPLC-PDA analysis were obtained from Derick Biotechnology Co., Ltd. (Chengdu, China). The standard phloretin was purchased from Ark Pharm, Inc. (Libertyville, IL, USA), and dihydromyricetin was obtained from Shanghai Acmec Biochemical Co., Ltd. (Shanghai, China). The distilled water was used in this study.

### 2.2. Kombucha Production

Kombucha starter culture was bought from Shandong Ruyun Edible Fungus Planting Co., Ltd. (Liaocheng, China), including tea fungus, cellulose pellicle, fermented broth and teabag (5 g black tea), and stored at 4 °C. The kombucha was produced based on a previous study with minor modifications [18], and carried out as the following steps: 1 L water and 100 g sucrose (i.e., 100 g/L of sucrose) were boiled, then added teabag (5 g black tea, i.e., 5 g/L of tea) into the mixture, and infused for 5 min. The teabag was removed, and the tea infusion was added into a sterilized conical flask. When the tea infusion was cooled to room temperature (25 °C), and the tea fungus, cellulose pellicle and fermented broth were added into conical flask, which was placed in a clean and dark place at room temperature for 14 days for subsequent inoculations.

### 2.3. Preparation of Kombucha with or without Tea Residues

The 300 mL distilled water was added into a 500 mL conical flask, and heated in a water bath until it was boiling, then 30 g of sucrose (i.e., 100 g/L of sucrose) was added. After the sucrose was completely dissolved, 3 g of vine tea or sweet tea (i.e., 10 g/L of vine tea or sweet tea) was added to the conical flask, and took out the conical flask immediately after 5 min, then cooled to room temperature. The mixture was filtered through a filter, and the infusion was collected for fermentation without tea residues. For fermentation with tea residues, the mixture was not filtered. The 30 mL of activated kombucha starter culture (i.e., 10% *v*/*v*) was added into the mixture. Therefore, four kombucha beverages included kombucha from vine tea without residues, kombucha from vine tea with residues, kombucha from sweet tea without residues, and kombucha from sweet tea with residues. Three parallel samples per group were prepared. Finally, these conical flask mouths were covered with a clean gauze and secured with rubber bands, then placed in a clean and dark place for fermentation at room temperature for 15 days. The samples were collected from conical flasks on days 0, 3, 6, 9, 12 and 15, and filtered through a 0.45 µm membrane for the measurement of antioxidant activities, TPC, and concentrations of bioactive compounds.

### 2.4. Antioxidant Capacity Assays

The assays of FRAP and TEAC were used to evaluate the antioxidant activities of kombucha beverages. These assays were performed based on the published literature [19,20,21].

The FRAP reaction solution was prepared by mixing 300 mmol/L sodium acetate buffer, 10 mmol/L TPTZ solution, and 20 mmol/L ferric chloride solution at a volume ratio of 10:1:1, then placed in a 37 ℃ water bath for subsequent experiments. The 100 µL diluted sample was added to 3 mL FRAP reaction solution for 4 min at room temperature. Finally, the absorbance of the mixture was measured at 593 nm with a spectrophotometer. The standard curves were made with different concentrations of ferrous sulfate solutions, and the FRAP values were shown as μmol Fe(II)/L.

The ABTS^•+^ stock solution was prepared with an equal volume of 7 mmol/L ABTS^•+^ solution and 2.45 mmol/L potassium persulfate solution, and stored in the dark for 16 h as well as used within 48 h. The ABTS^•+^ stock solution was diluted with distilled water that made its absorbance of 0.71 ± 0.05 at 734 nm and configured as ABTS^•+^ reaction solution. The 100 µL diluted sample was added to 3.8 mL ABTS^•+^ reaction solution, and then the mixture was incubated at room temperature for 6 min in the dark. Finally, the absorbance of the mixture was measured at 734 nm with a spectrophotometer. The standard curves were made using different concentrations of Trolox solutions, and the TEAC values were shown as μmol Trolox/L.

### 2.5. Measurement of Total Phenolic Content

The TPC was determined using the Folin-Ciocalteu method based on previous literature [21]. The 500 µL diluted sample was added to 2.5 mL 0.2 mol/L Folin-Ciocalteu reagent and reacted for 4 min, and then 2 mL saturated sodium carbonate solution (75 g/L) was added. The mixture was incubated at room temperature for 2 h in the dark, and then the absorbance of the mixture was determined at 760 nm with a spectrophotometer. The different concentrations of gallic acid were applied to make the standard curves, and the TPC values were expressed as mg of gallic acid equivalent (GAE)/L.

### 2.6. Analysis of the Concentrations of Bioactive Components in Kombucha Beverages

The concentrations of bioactive components in kombucha beverages were measured by HPLC-PDA based on previous studies [12,17]. Separation was performed using an Agilent Zorbax Eclipse XDB-C18 column (250 mm × 4.6 mm, 5 µm) (Santa Clara, CA, USA). The column temperature was 35 °C, the sample chamber temperature was 4 °C, and the flow rate was 0.8 mL/min with the injection volume of 20 μL. The mobile phase constituted of solution A (methanol) and solution B (0.1% formic acid), and the elution procedure is shown in Table 1. The bioactive compounds in kombucha beverages were identified by comparison with the retention time and UV-visible spectrum of the standards, and their concentrations were quantified by the peak area at the maximum absorption wavelength, and the concentrations were expressed as mg/L.

### 2.7. Sensory Analysis

The sensory analysis of kombucha beverages was carried out based on the previous study [22]. The kombucha beverages were scored by 8 panelists from the Department of Nutrition, School of Public Health, Sun Yat-sen University. The odour, color, flavor, sourness, and overall acceptability of the kombucha beverages were rated on a scale from 1–9, and the scoring criteria are shown in Table 2.

### 2.8. Statistical Analysis

The experimental data were shown as mean ± standard deviation. The SPSS 25.0 statistical software (IBM Corp., Armonk, NY, USA) and Excel 2016 (Microsoft, Washington, DC, USA) were used to analyze the antioxidant activities, TPC and HPLC-PDA results. The statistical significance was analyzed by one-way analysis of variance (ANOVA), and defined at *p* < 0.05. The correlations between parameters and concentrations of compounds were calculated from Pearson correlation index using the SPSS 25.0 statistical software, and the heatmaps were carried out by https://www.chiplot.online on 12 August 2022.

## 3. Results and Discussion

**Figure 1:**
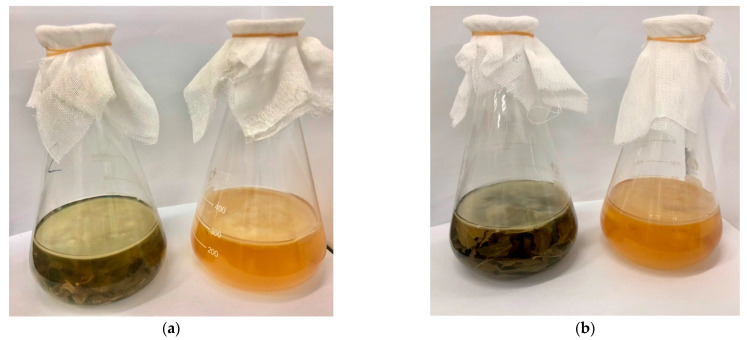
The appearance of kombucha beverages fermentation with or without tea residues. (**a**) kombucha from vine tea with or without tea residues; (**b**) kombucha from sweet tea with or without tea residues.

Kombucha beverages are rich in various bioactive components, and have many health benefits. Numerous studies have shown that alternative raw materials could allow the kombucha beverages significant antioxidant activities and increase the amounts of bioactive compounds [10,23,24]. Moreover, fermentation with tea residues was found to increase antioxidant capacities as well as polyphenol contents of kombucha [12]. Therefore, in this study, we adopted vine tea and sweet tea to develop new kombucha beverages, and compared the effects of kombucha beverages fermentation with or without tea residues. The kombucha beverages prepared with or without tea residues are shown in Figure 1.

### 3.1. Antioxidant Capacities of Kombucha

#### 3.1.1. FRAP Values

**Figure 2:**
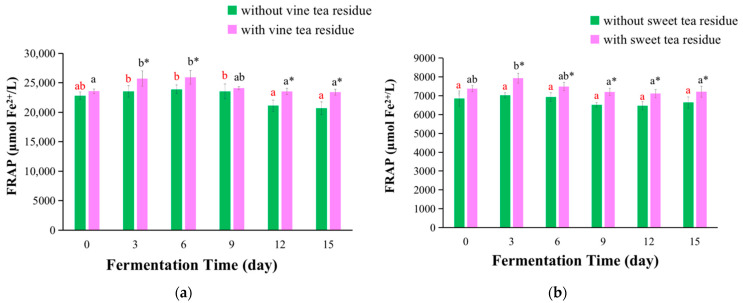
Changes of FRAP values of kombucha beverages at different fermentation time. (**a**) FRAP values of kombucha from vine tea; (**b**) FRAP values of kombucha from sweet tea. The different red letters indicated that kombucha fermentation without tea residues at different fermentation time were significant differences (*p* < 0.05), and the same red letter indicated no significant difference (*p* > 0.05). The different black letters indicated that kombucha fermentation with tea residues at different fermentation time had significant differences (*p* < 0.05), and the same black letter indicated no significant difference (*p* > 0.05). * Indicated that there was a significant difference between the kombucha fermentation with tea residues and kombucha fermentation without tea residues at the same fermentation time (*p* < 0.05).

The FRAP assay was a method to assess the reducing power of Fe^3+^ to Fe^2+^, and was widely applied to determine the antioxidant activities of fruits, vegetables, and medicinal herbs [25]. The FRAP values of kombucha fermentation with or without vine tea residues and sweet tea residues are shown in Figure 2.

The FRAP values of vine tea kombucha are presented in Figure 2a. For vine tea kombucha with tea residues, with the prolonging of fermentation time, the FRAP values first increased and then reduced, and reached the maximum on day 6. For vine tea kombucha without tea residues, the FRAP values did almost not change before day 9, and then decreased. Additionally, the FRAP values of kombucha fermented with tea residues were higher compared with those without tea residues, which increase 9% on day 3 (Figure 2a).

The FRAP values of sweet tea kombucha are presented in Figure 2b, which reached the maximum values on day 3 and then decreased with the prolonging of fermentation time for kombucha with tea residues. For sweet tea kombucha without tea residues, the FRAP values did almost not change during fermentation time. Moreover, the FRAP values of kombucha fermented with tea residues were markedly higher (increasing 13% on day 3) than those without tea residues (Figure 2b).

The fermentation with tea residues increases the FRAP values of kombucha beverages, and sweet tea residues expressed slightly higher effect than vine tea residues. A previous study showed that the FRAP values of kombucha prepared with green tea residues was as 3.13 times as that of without tea residues [12]. However, in this paper, the FRAP values of kombucha prepared with tea residues of vine tea and sweet tea increased less than that of kombucha from green tea. This could be because the cells of vine tea and sweet tea leaves were disrupted more serious than those of green tea leaves during the produce process of teas. Therefore, the compounds in vine tea and sweet tea are easier dissolved in water than those in green tea during the preparation of kombucha fermentation infusion, just like the difference of green tea and black tea [12]. This would result in a lower content of compounds in vine tea and sweet tea residues than in green tea residues, and the compounds in green tea residues could be further dissolved in broth under the action of enzymes in kombucha production process. Additionally, the FRAP values of kombucha beverages in this study were significantly higher than those of kombucha from black tea, white tea, red tea, and green tea (2725.9 ± 41.0, 3263.8 ± 46.3, 4314.3 ± 53.5, and 4801.1 ± 69.2 μmol Fe(II)/L, respectively) reported in the literature [26].

#### 3.1.2. TEAC Values

**Figure 3:**
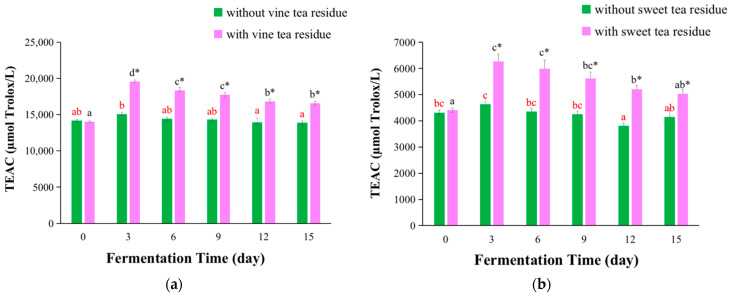
Changes of TEAC values of kombucha beverages at different fermentation time. (**a**) TEAC values of kombucha from vine tea; (**b**) TEAC values of kombucha from sweet tea. The different red letters indicated that kombucha fermentation without tea residues at different fermentation time were significant differences (*p* < 0.05), and the same red letter indicated no significant difference (*p* > 0.05). The different black letters indicated that kombucha fermentation with tea residues at different fermentation time had significant differences (*p* < 0.05), and the same black letter indicated no significant difference (*p* > 0.05). * Indicated that there was a significant difference between the kombucha fermentation with tea residues and kombucha fermentation without tea residues at the same fermentation time (*p* < 0.05).

The TEAC assay is to measure the scavenging ability of compounds against ABTS∙^+^ radical cations, and the TEAC value can be determined by comparing the scavenging ability of all compounds with that of Trolox [27]. The TEAC assay has been applied to measure the antioxidant capacity of various phytochemicals and synthetic compounds [28,29]. The TEAC values of kombucha fermentation with or without vine tea residues and sweet tea residues are shown in Figure 3.

The TEAC values of vine tea kombucha are illustrated in Figure 3a. The TEAC values increased at first and then decreased with the extension of fermentation time, and reached the maximum value on day 3. Visibly, the TEAC values of kombucha with tea residues were significantly higher compared with those of kombucha without tea residues, which was increased 30% on day 3 (Figure 3a). The TEAC values of vine tea kombucha with or without tea residues in this study were higher compared with that of kombucha from black tea in the literature (4634.4 μmol Trolox/L) [30].

The TEAC values of sweet tea kombucha are presented in Figure 3b. The change trend of TEAC values of sweet tea kombucha was similar to that of vine tea kombucha. In addition, the TEAC values of kombucha with tea residues was significantly higher compared with those of kombucha without tea residues, which was increased 38% on day 6 (Figure 3b). The TEAC values of sweet tea kombucha with tea residues in this study were generally higher compared with that of kombucha from black tea (4634.4 μmol Trolox/L) in a previous study, while the TEAC values without tea residues were slightly lower [30].

The fermentation with tea residues could significantly improve the TEAC values of kombucha beverages, and the effect of sweet tea residues was slightly better than that of vine tea residues, which was similar to the FRAP results. The difference could be induced by several factors. For example, extraction time could influence the extraction efficiency of bioactive components from leaves. In this paper, the leaves were boiled for 5 min, and cooled to room temperature which cost about 30 min. That is, total extraction time was about 35 min. This was a longer time than those in previous studies, where the extraction time was generally 5 min using boiling water with immediate filtration [31,32,33]. In addition, a single extraction was very hard to obtain all bioactive components from leaves using boiling water according to our previous studies [34,35]. Thus, some bioactive components could still be kept in leaves, and could be dissolved into the broth, which would result in that kombucha with tea residue had a higher TEAC value.

### 3.2. TPC Values of Kombucha

**Figure 4:**
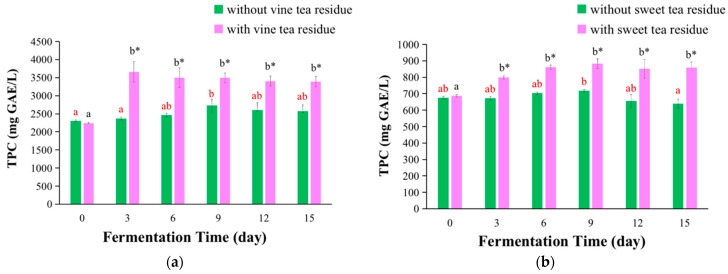
Changes of TPC values of kombucha beverages at different fermentation time. (**a**) TPC values of kombucha from vine tea; (**b**) TPC values of kombucha from sweet tea. The different red letters indicated that kombucha fermentation without tea residues at different fermentation time were significant differences (*p* < 0.05), and the same red letter indicated no significant difference (*p* > 0.05). The different black letters indicated that kombucha fermentation with tea residues at different fermentation time had significant differences (*p* < 0.05), and the same black letter indicated no significant difference (*p* > 0.05). * Indicated that there was a significant difference between the kombucha fermentation with tea residues and kombucha fermentation without tea residues at the same fermentation time (*p* < 0.05).

The Folin-Ciocalteu method has been widely used to determine TPC in numerous studies because of its simple, rapid, and inexpensive [34,35]. The TPC values of kombucha fermentation with or without vine tea residues and sweet tea residues are presented in Figure 4.

The TPC values of vine tea kombucha are exhibited in Figure 4a. The TPC values increased at first and then slightly decreased with the extension of the fermentation time. The maximum values of kombucha with or without tea residues were arrived on days 3 and 9, respectively. Furthermore, the TPC values of kombucha with tea residues were significantly higher compared with those without tea residues, which was increased 55% on day 3 (Figure 4a).

The TPC values of sweet tea kombucha are exhibited in Figure 4b. With the extension of fermentation time, the change trend of TPC values of sweet tea kombucha was firstly increased and then reduced, and the TPC values arrived the maximum value on day 9. Furthermore, the TPC values of kombucha with tea residues were markedly higher compared with those without tea residues, with an increase of 35% on day 15 (Figure 4b).

The fermentation with tea residues could significantly improve the TPC values of kombucha beverages, and the effect of vine tea residues was stronger than that of sweet tea residues. The differences could be induced by various factors, which were similar to those of TEAC values as mentioned above. Additionally, the TPC values of kombucha beverages in this study were significantly higher than those of kombucha from black tea, white tea, red tea, green tea, kitchen mint, oolong tea, and snake fruit (219.9 ± 2.1, 228.1 ± 0.5, 271.9 ± 3.6, 320.1 ± 3.5, 463.33 ± 5.02, 485.18 ± 1.72, and 535.59 ± 1.96 mg GAE/L, respectively) reported in the literature [9,26,36]. It could be partly because that vine tea and sweet tea had higher TPC values than those of most plants reported in the literature [37,38,39].

### 3.3. Concentrations of Bioactive Components in Kombucha

**Figure 5:**
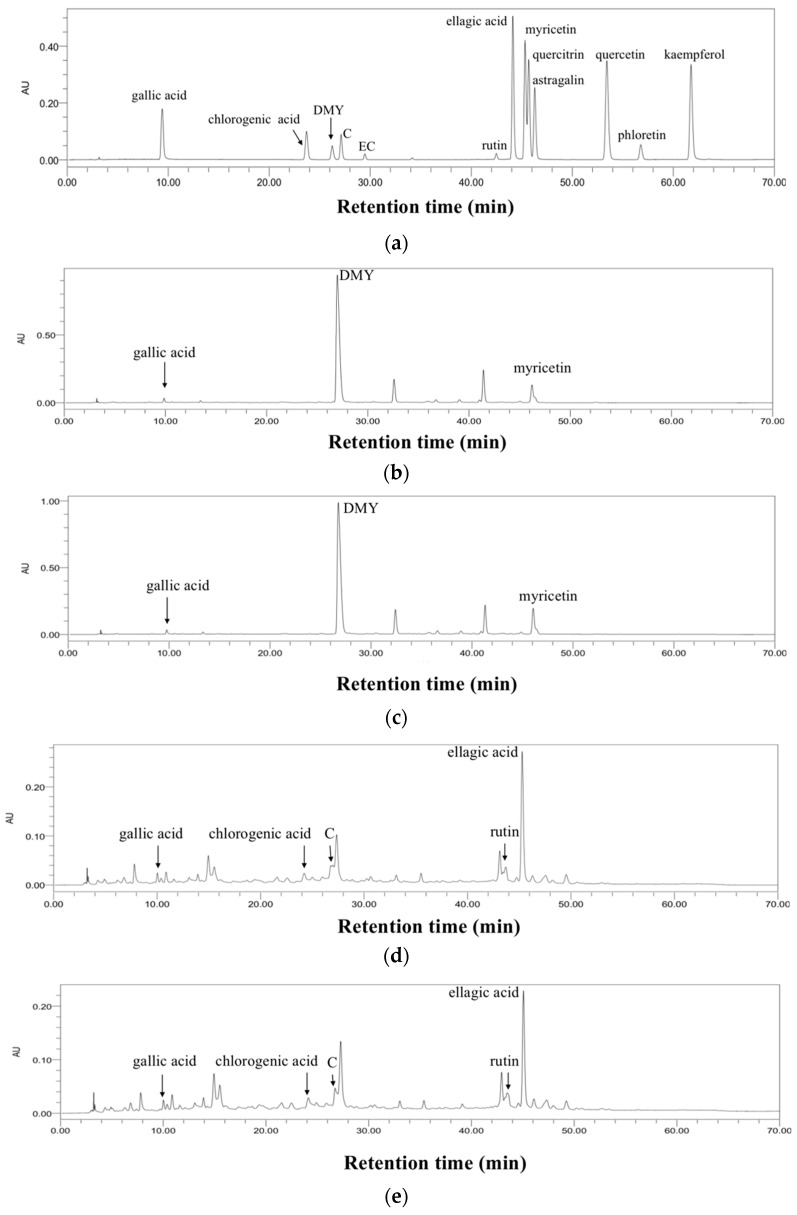
The representative chromatograms of standards and kombucha beverages at 254 nm. (**a**) standards, (**b**) kombucha from vine tea without tea residues, (**c**) kombucha from vine tea with tea residues, (**d**) kombucha from sweet tea without tea residues, and (**e**) kombucha from sweet tea with tea residues. DMY, dihydromyricetin; C, catechin; EC, epicatechin.

The bioactive components in kombucha beverages were separated and quantified by HPLC-PDA, and representative chromatograms are exhibited in Figure 5. Three components were picked out from vine tea kombucha, including gallic acid, dihydromyricetin (DMY), and myricetin (Figure 5b,c). Moreover, five compounds were identified from sweet tea kombucha, including gallic acid, chlorogenic acid, catechin, rutin, and ellagic acid (Figure 5d,e).

**Figure 6:**
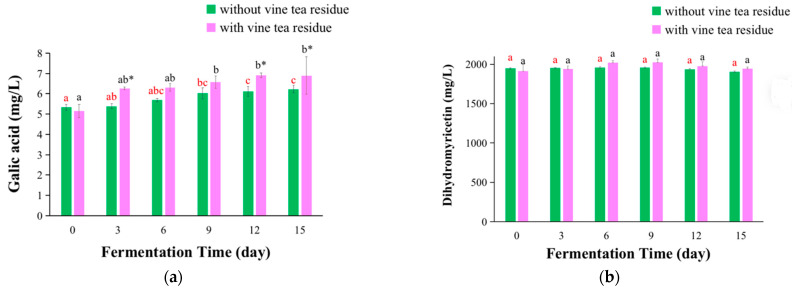

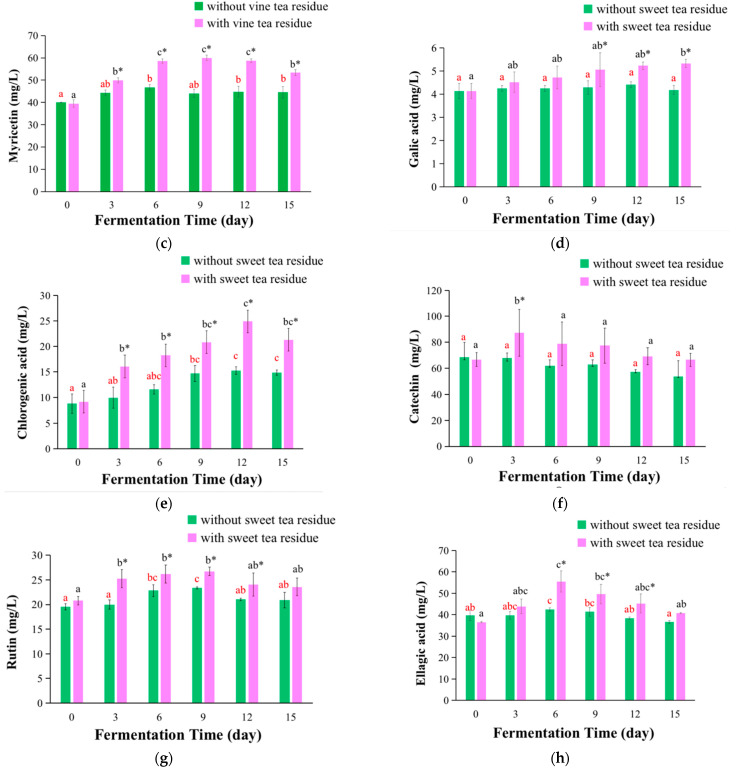
The changes of concentrations of components of kombucha produced by fermentation with or without tea residues at different fermentation time. (**a**–**c**) kombucha from vine tea, and (**d**–**h**) kombucha from sweet tea. The different red letters indicated that kombucha fermentation without tea residues at different fermentation time were significant differences (*p* < 0.05), and the same red letter indicated no significant difference (*p* > 0.05). The different black letters indicated that kombucha fermentation with tea residues at different fermentation time had significant differences (*p* < 0.05), and the same black letter indicated no significant difference (*p* > 0.05). * Indicated that there was a significant difference between the kombucha fermentation with tea residues and kombucha fermentation without tea residues at the same fermentation time (*p* < 0.05).

The peak area under the maximum absorption wavelength was applied for the quantification of components in kombucha beverages, and the results are shown in Figure 6. The concentrations of gallic acid and myricetin in kombucha fermented with vine tea residues were higher compared with those of kombucha without tea residues (Figure 6a,c). This was because that these components could be continuously dissolved to the broth from tea residues, which were similar to those of kombucha from black tea and green tea [12]. Moreover, it was noted that kombucha from vine tea contained a very high concentration of DMY (Figure 6b). Although there was no significant difference, the concentration of DMY in kombucha fermented with vine tea residues was higher than those of kombucha without tea residues after day 6 (Figure 6b).

For the kombucha prepared with sweet tea, the concentrations of the five compounds were all higher in kombucha with tea residues compared with those without tea residues (Figure 6d–h). The concentration of gallic acid in kombucha with residues was increased with the fermentation time (Figure 6d). This might be because that gallic acid were continuously dissolved to the broth from tea residues, which was similar to that of kombucha from black tea and green tea [12]. Furthermore, the concentration of gallic acid in kombucha without residues was basically unchanged (Figure 6d), which was very different from that of kombucha from black tea and green tea without tea residues, where the concentration of gallic acid was enhanced with the fermentation stages [12]. This was because that other compounds from black tea and green tea could be degraded to produce it, which was also in agreement with that in the literature, where the content of gallic acid was significantly increased when the green tea extract was enzymatically degraded using tannase [20]. Moreover, the concentration of catechin was reduced with the prolonging of fermentation stages in the kombucha without tea residues, which might be caused by catechin degradation under the action of microorganisms in the kombucha beverages. For fermentation with tea residues, the concentration of catechin increased at first and then decreased with the fermentation stages, which might be because catechin was dissolved to the broth from tea residues, and partly degraded by microorganisms in the broth (Figure 6f). Additionally, the concentrations of chlorogenic acid, rutin, and ellagic acid in kombucha with or without tea residues all showed a trend of first rising and then reducing with the fermentation time (Figure 6e,g,h), which was similar to catechin in kombucha fermentation with tea residues.

### 3.4. Correlations Analysis between Parameters and Concentrations of Compounds

**Figure 7:**
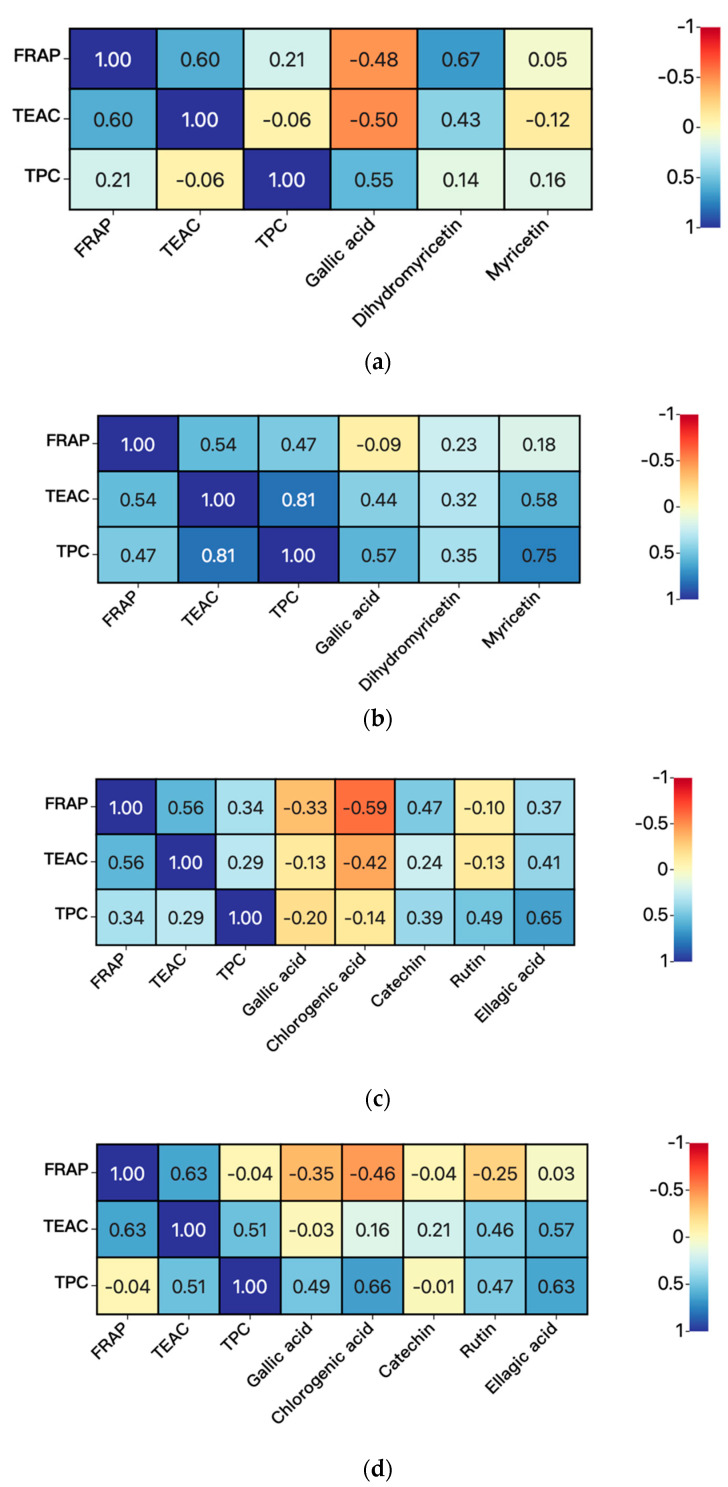
Heat maps analysis of the correlations between parameters and concentrations of compounds. (**a**) kombucha from vine tea without tea residues, (**b**) kombucha from vine tea with tea residues, (**c**) kombucha from sweet tea without tea residues, and (**d**) kombucha from sweet tea with tea residues.

The results of the heat maps analysis of the correlation among FRAP, TEAC, TPC, and concentrations of compounds are shown in Figure 7.

For FRAP and TEAC values, they were significantly correlations when the kombucha was prepared by fermentation without vine tea residues (R = 0.60) or with sweet tea residues (R = 0.63), which indicated that antioxidants in kombucha could reduce oxidants (e.g., Fe^3+^), and also capture free radicals [40]. Moreover, TEAC values were significantly related with TPC values in kombucha prepared by fermentation with vine tea residues (R = 0.81), which showed that phenolics in kombucha could contribute to the TEAC values [41].

For the relationships between FRAP values and concentrations of bioactive compounds: A significant correlation was obtained between FRAP values and concentration of DMY (R = 0.67) in kombucha without vine tea residues, which indicated that DMY in kombucha could contribute to the FRAP values. Moreover, the correlations between TEAC values and concentrations of bioactive components were no significant.

For the relationships between TPC values and concentrations of bioactive compound: A significant correlation was observed between TPC values and concentration of myricetin (R = 0.75) in kombucha with vine tea residues. In addition, a significant correlation was obtained between TPC values and concentration of ellagic acid (R = 0.65) in kombucha without sweet tea residues. Furthermore, the significant relations were existed between TPC values and concentrations of chlorogenic acid (R = 0.66) and ellagic acid (R = 0.63) in kombucha with sweet tea residues, which indicated that these compounds in kombucha could contribute to the TPC values.

### 3.5. Sensory Properties of Kombucha

**Figure 8:**
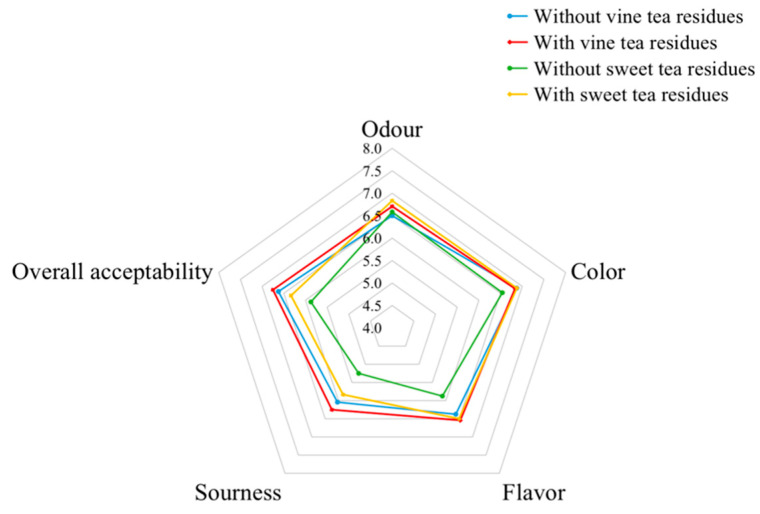
Sensory property of kombucha beverages.

Sensory evaluations of the kombucha with or without tea residues were performed for odour, color, flavor, sourness, and overall acceptability, and the results are shown in Figure 8. Generally, kombucha beverages fermented with vine tea residues or sweet tea residues had higher scores or no significant differences for these sensory properties compared with kombucha without tea residues, which showed that the sensory properties of kombucha beverages fermentated with tea residues were acceptable with higher antioxidant activities and TPC. Furthermore, the kombucha beverages from vine tea had higher scores compared with those of kombucha beverages from sweet tea, and also had higher antioxidant activities and TPC, which showed that vine tea kombucha was generally a better beverage than sweet tea kombucha.

## 4. Conclusions

In this paper, kombucha beverages from vine tea and sweet tea are studied for the first time, and influences of tea residues on antioxidant activities as well as TPC are evaluated. The results indicated that fermentation with tea residues could significantly enhance FRAP, TEAC as well as TPC values of kombucha beverages, which had also higher scores or no significant differences for several sensory properties compared with kombucha without tea residues. In addition, the bioactive compounds in kombucha beverages were separated and quantified by HPLC-PDA, and the change trends of concentrations of several compounds were different with the prolonging of fermentation time. Generally, fermentation with tea residues could rise the concentrations of several compounds. Therefore, the production of kombucha beverages by fermentation with tea residues from non-*Camellia sinensis* plants (vine tea and sweet tea) is very promising because of their pleasant sensory properties and high antioxidant activities, which could be potential to be used as functional food to prevent several diseases caused by oxidative stress.

## Figures and Tables

**Table 1:** The procedure of gradient elution.

Time (Min)	Gradient Elution
0–10	2–17% A
10–15	17–19% A
15–20	19–22% A
20–40	22–47% A
40–50	47–50% A
50–60	50–58% A
60–70	58–2% A
70–75	2% A

**Table 2:** Scoring criteria for sensory evaluation.

Score	Represent
1	Extreme disliking
2	Great disliking
3	Moderate disliking
4	Slight disliking
5	Neither liking nor disliking
6	Slight liking
7	Moderate liking
8	Great liking
9	Extreme liking

## Data Availability

Data is contained within the article.
